# Tumor Hypoxia Heterogeneity Affects Radiotherapy: Inverse-Percolation Shell-Model Monte Carlo Simulations

**DOI:** 10.3390/e24010086

**Published:** 2022-01-05

**Authors:** Argyris Dimou, Panos Argyrakis, Raoul Kopelman

**Affiliations:** 1 Department of Physics and Complexity Center, University of Thessaloniki, 54124 Thessaloniki, Greece; ardimou@physics.auth.gr; 2 Department of Chemistry, University of Michigan, Ann Arbor, MI 48109, USA; kopelman@umich.edu

**Keywords:** hypoxia heterogeneity, tumor radiotherapy, inverse percolation shell model monte-carlo simulations, oncology radiation modelling

## Abstract

Tumor hypoxia was discovered a century ago, and the interference of hypoxia with all radiotherapies is well known. Here, we demonstrate the potentially extreme effects of hypoxia heterogeneity on radiotherapy and combination radiochemotherapy. We observe that there is a decrease in hypoxia from tumor periphery to tumor center, due to oxygen diffusion, resulting in a gradient of radiative cell-kill probability, mathematically expressed as a probability gradient of occupied space removal. The radiotherapy-induced break-up of the tumor/TME network is modeled by the physics model of inverse percolation in a shell-like medium, using Monte Carlo simulations. The different shells now have different probabilities of space removal, spanning from higher probability in the periphery to lower probability in the center of the tumor. Mathematical results regarding the variability of the critical percolation concentration show an increase in the critical threshold with the applied increase in the probability of space removal. Such an observation will have an important medical implication: a much larger than expected radiation dose is needed for a tumor breakup enabling successful follow-up chemotherapy. Information on the TME’s hypoxia heterogeneity, as shown here with the numerical percolation model, may enable personalized precision radiation oncology therapy.

## 1. Introduction

A tumor and its tumor micro-environment (TME) usually have a center, a periphery, and a density gradient in-between. Tumor center: Whether at a tumor’s original location, at a colony due to metastasis, or in a xenograft animal model due to implantation, tumors usually have a center-of-mass made of tumor cells. These cells extend out to a periphery, which may be well-defined or fuzzy, with a “clean” or fractal-like boundary. There is also usually a density gradient between the center and the periphery. Here, we study models that mimic those properties. Furthermore, while these models may primarily apply to the “physical” mass distribution of the tumor cells, they may also apply to some other property of the TME, such as its chemical component distribution, e.g., its acidity (increase of H^+^ or lowering of pH), oxygen content (depletion of O_2_ or hypoxia), or extracellular potassium ions (excess of K^+^ or hyperkalemia) [1]. The understanding of such a mass distribution, density gradient, or connectivity network may have important implications for understanding the tumor biology and for its medical treatment. Notably, both therapy and imaging are often affected by tumor penetration difficulty, either by the drug or by the imaging contrast agent [2]. As an example, treatment by chemotherapy may be optimal only at the tumor periphery, because the progress of the drug into the tumor center may be hampered by the TME’s acidity. In addition, the hypoxia, i.e., O_2_ concentration depletion, at the center of a tumor is likely to differ from that at its periphery. Such depletion, while always reducing the effectivity of any radiation therapy, may also affect its combination with chemotherapy, as discussed below. Similarly, the distribution of the TME’s hyperkalemia may affect the success of immunochemistry, but this will be discussed elsewhere. The Monte Carlo simulations presented here are aimed at studying the consequences of such density gradients on the network’s break-up, whether related to the network of the tumor cells, the TME’s acidity (pH), or its O_2_ concentration. Notably, the information on such distributions may guide therapy options, i.e., whether chemotherapy, radiation therapy, combination therapy, or surgery would be the best route. Here, we limit the discussion to combination therapy involving radiation as the first step, followed by chemotherapy. This discussion may also rationalize existing protocols derived empirically [3].

Historically the earliest, and still most common, tumor treatment employs drugs, i.e., chemotherapy [3]. Notably, the drug doses are limited by their notorious side effects. However, tumors tend to resist chemotherapy by using one of their “chemical weapons”, specifically, the acidity of the TME [4,5,6,7,8]. This “acidosis” (low pH) of the TME has been discovered over a century ago by Warburg [9]. Most drug molecules may decompose due to such acidity. Thus, currently, tumor treatment often starts with radiation therapy, followed by chemotherapy [3]. Presumably the rationale is to break up the extended network of the tumor cells, and their TMEs, into isolated “clusters”. Such break-up would enable some of the drug molecules to avoid the acidic TMEs, and thus stay intact until reaching the tumor cells, and eventually kill them. A mathematical model describing such break-up of the tumor network, or of its TME network, is the main topic of this study. We also derive pictures illustrative of the tumor tissue, and its TMEs, after varying radiation doses, under a variety of simulated hypoxia anisotropy conditions.

In a randomized two-component lattice model, the formation of an extended network of one given component was studied mathematically first by Hammersley, in terms of a percolation model [10,11]. The latter describes highly nonlinear, i.e., catastrophic, behavior, e.g., phase transitions in physics. Geometrically, it describes the break-up of some connected network. The break-up of such an extended network is mathematically equivalent to its formation process and is thus called inverse percolation [12]. Either the network formation or its break-up occur at a “critical concentration” of the relevant component. This “critical concentration” has occasionally been derived analytically, but mostly requires the use of Monte Carlo simulations [10,11]. We here consider a tissue lattice made of two components, live and dead tumor cells, where the dead cells are the result of radiation therapy. As the radiation kills cells randomly, at least to first approximation, such a percolation model should be appropriate. However, there is an additional consideration. Tumors also have a “chemical weapon” against radiation therapy: Hypoxia, the absence of tissue oxygen. Again, the low concentration of oxygen in the TME has been known over a century, due to Warburg [9]. The hypoxia is the result of the tumor cells’ enhanced metabolism, due to their accelerated growth and multiplication. We note, however, that the chemical mechanism of cell-kill by radiation relies on the presence of O_2_ molecules. Specifically, the radiation energy excites the O_2_ molecules from their “triplet” ground state into their higher energy “singlet” state. “Singlet oxygen” has been called “killer oxygen”, as it produces the so-called “reactive oxygen species” (ROS) that kill cells [13]. One typical ROS member is the OH radical molecule; another is the singlet oxygen molecule. Notably, the oxygen depletion will be highest at the tumor’s center and lowest at its periphery, where the oxygen molecules are replenished by diffusion from the nearby, oxygen-rich, normal tissue. We thus employ a model where the radiation cell-kill may be most effective at the tumor’s outer shell (periphery) and least effective at its center. This gives rise to a new percolation model, employing a lattice with shells of different probability regimes. We thus apply an originally random distribution of tumor cells, with a shell-to-shell density gradient of kill probability. As a first step towards illustrating this approach, we use a simple two-dimensional “onion-like” shelled lattice model. We show that the “critical concentration” for the live tumor cell network break-up is higher than for a normal lattice. The potential ramifications for radiation and chemotherapy are discussed. We give graphical illustrations of our preliminary insights regarding radiotherapy efficacy. These insights still need to be tested in a computationally more intensive three-dimensional model. In the future they may be tested on specific geometries characterizing specific tumors and their TMEs, thus enabling personalized precision radiation oncology therapy.

## 2. Method of Simulations

The system we use is a square lattice, where each site has four (4) nearest neighbors. This system has been well studied in the context of the percolation problem, in which the lattice has “filled” and “open” sites, randomly distributed, with a concentration (probability) p of filled sites. This system has been shown to undergo a higher-order phase transition at the “critical point” p_c_ = 0.5927. Usually, one starts with an empty lattice and starts filling the sites randomly until the critical point is reached, which is the point where, for the first time, a spanning cluster (connected network) appears that is connected throughout the lattice from one end to the other [10,11]. In our case, we work with the inverse problem, i.e., we start with a fully occupied (filled) lattice, taking out sites randomly, until the same critical point is reached; in this case, it is the point where, for the first time, the spanning cluster disappears, so that the ends of the lattice are not connected anymore. This has been called “inverse percolation”.

**Figure 1:**
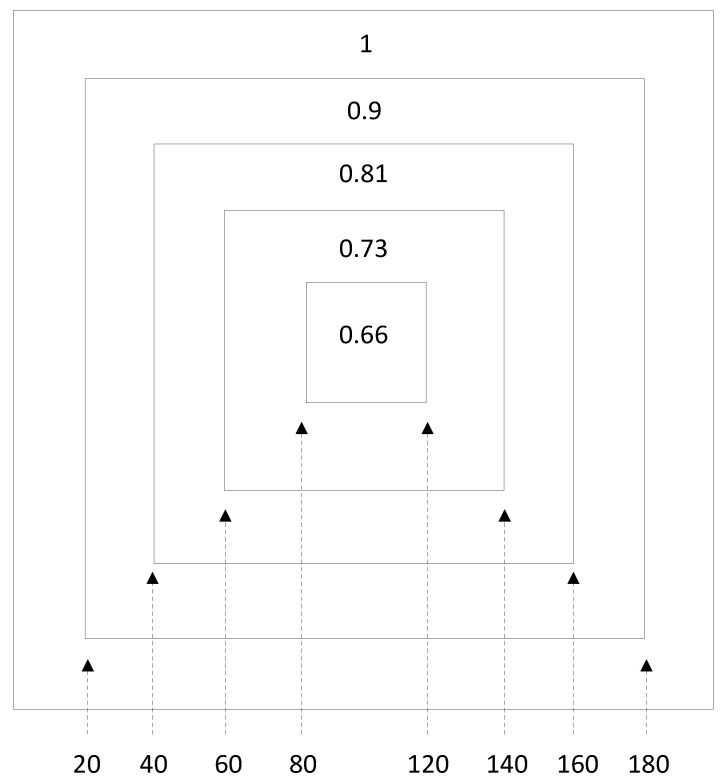
Division of a 200 × 200 lattice into shells. We show the probabilities of removal for every zone for r = 0.1.

In this work, the new objective is a case where the sites are not removed with equal probability, in contrast to the classical case, so as to reach the critical point, but instead they are removed, in each shell of the lattice, with different removal probabilities; specifically, with a higher probability, the closer they are to the periphery of the lattice, and with a lower probability, the closer they are to the center of the lattice. Thus, the lattice is divided into several shells (zones), each shell having a different probability for the removal of a site. An example is shown in Figure 1, where the lattice is divided into five (5) square shells. Each site of the lattice belongs to one of those shells, according to its distance from the center. The lattice shown in Figure 1 is of size 200 × 200, and the boundaries of the five shells are marked. Inside each shell is denoted the probability of removal of a site, when chosen. Using a uniform random number distribution, we select an occupied (filled) site of the lattice. That site will be removed with probability denoted in the corresponding shell. If the site is not taken out then a new site is chosen. Whenever the site is removed, we check with the CMLT algorithm [14] whether the largest cluster percolates. The criterion used is that sites at all ends of the lattice, either up/down or right/left, belong to its largest cluster, i.e., are all connected. Thus, in the current work we remove sites one-by-one and we identify the critical point exactly when the largest cluster seizes to percolate. We then calculate the density of the still-remaining occupied sites, which gives us the percolation threshold of this Monte Carlo realization. This procedure for sequentially removing occupied sites with a certain probability is repeated until the critical point is reached. In the present case, where the probabilities for removal are different, we expect the p_c_ to differ from the “normal” (non-shelled square lattice) percolation threshold (p_c_ = 0.5927) [10].

## 3. Mathematical Results

**Figure 2:**
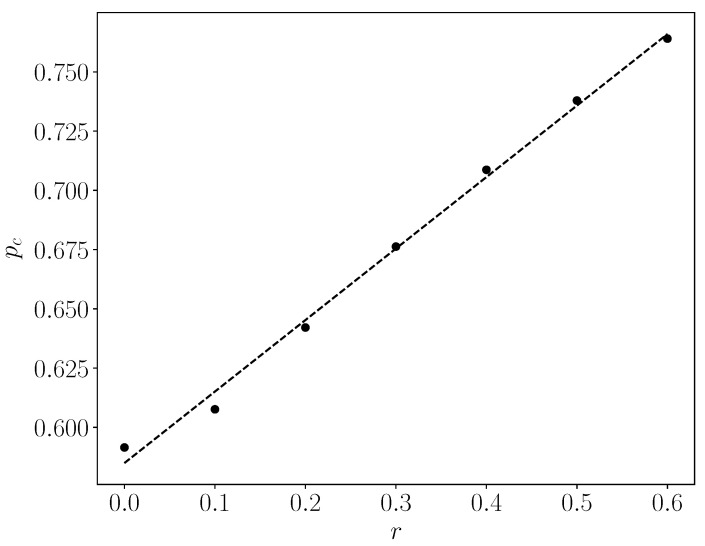
Percolation threshold, p_c_, of a 1000 × 1000 square lattice as a function of r, the rate of reduction of the probability for removal from one shell to its next shell, for 100 realizations.

In Figure 2, we plot the percolation threshold p_c_ of the lattice as a function of r. We define the parameter r as the rate of reduction of the probability for removal from one shell to its next shell towards the center. We set the probability for removal of a site in the outer shell as 1. Thus, for r = 0.1, and for the case of five shells, the list of probabilities for removal in the five shells will be p = [0.66, 0.73, 0.81, 0.9, 1], where 0.66 is the probability of the innermost shell, and 1 is the probability of the outermost shell. See the distribution of these probabilities in the different shells in Figure 1.

As expected, as r increases, the lattice percolates at larger values of p_c_. This is reasonable, as in our algorithm many attempts of removing a random closed site of an inner shell will fail, resulting in having fewer inner sites and more outer sites being removed. Thus, the maximal (all connected) cluster will likely stop existing in the periphery, as a result of having removed more sites from there. We observe that for r = 0, p_c_ = 0.5915, which agrees within error with the known critical value of 0.5927. We also see that there is a linear increase of p_c_ with r. The best fit of the straight line is shown in the figure and has a slope 0.315. Table 1 contains the critical threshold values p_c_ as a function of r.

**Figure 3:**
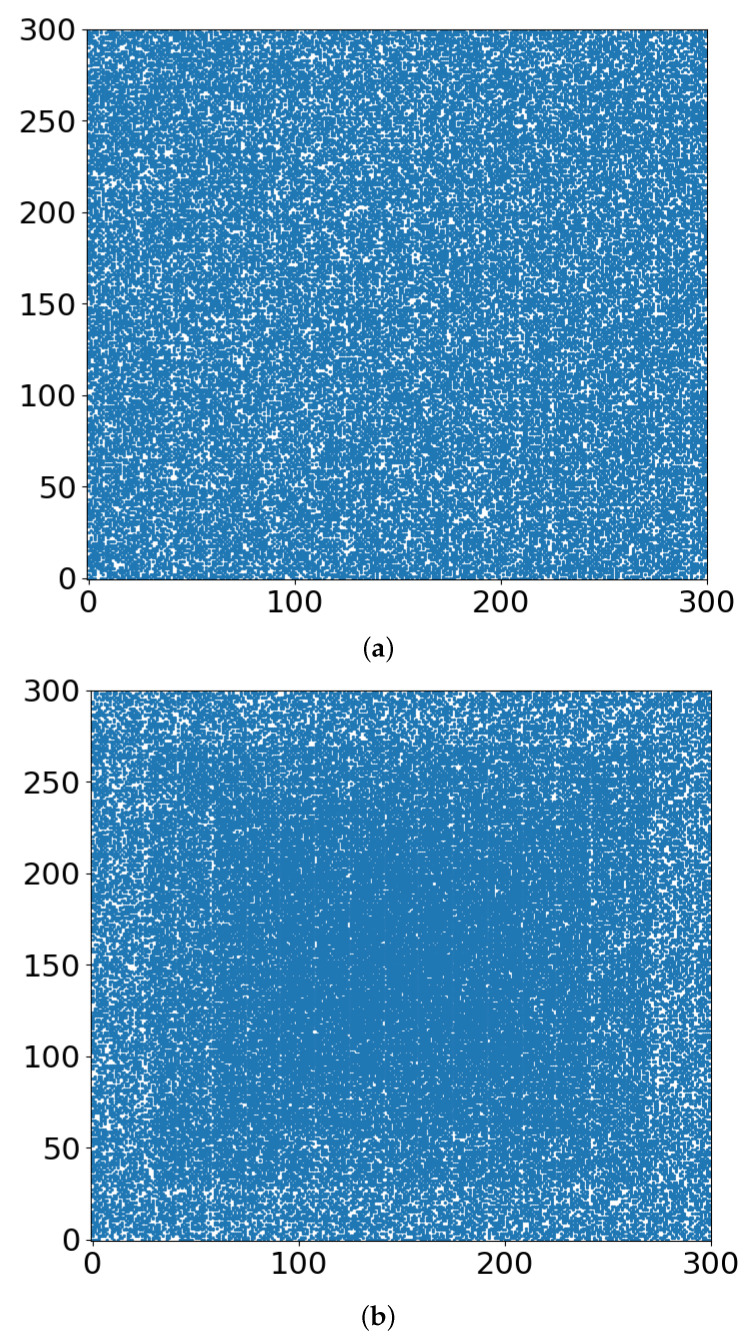
Occupied sites of the lattice (blue dots) when the largest cluster percolates for (**a**) r = 0, (**b**) r = 0.4.

**Figure 4:**
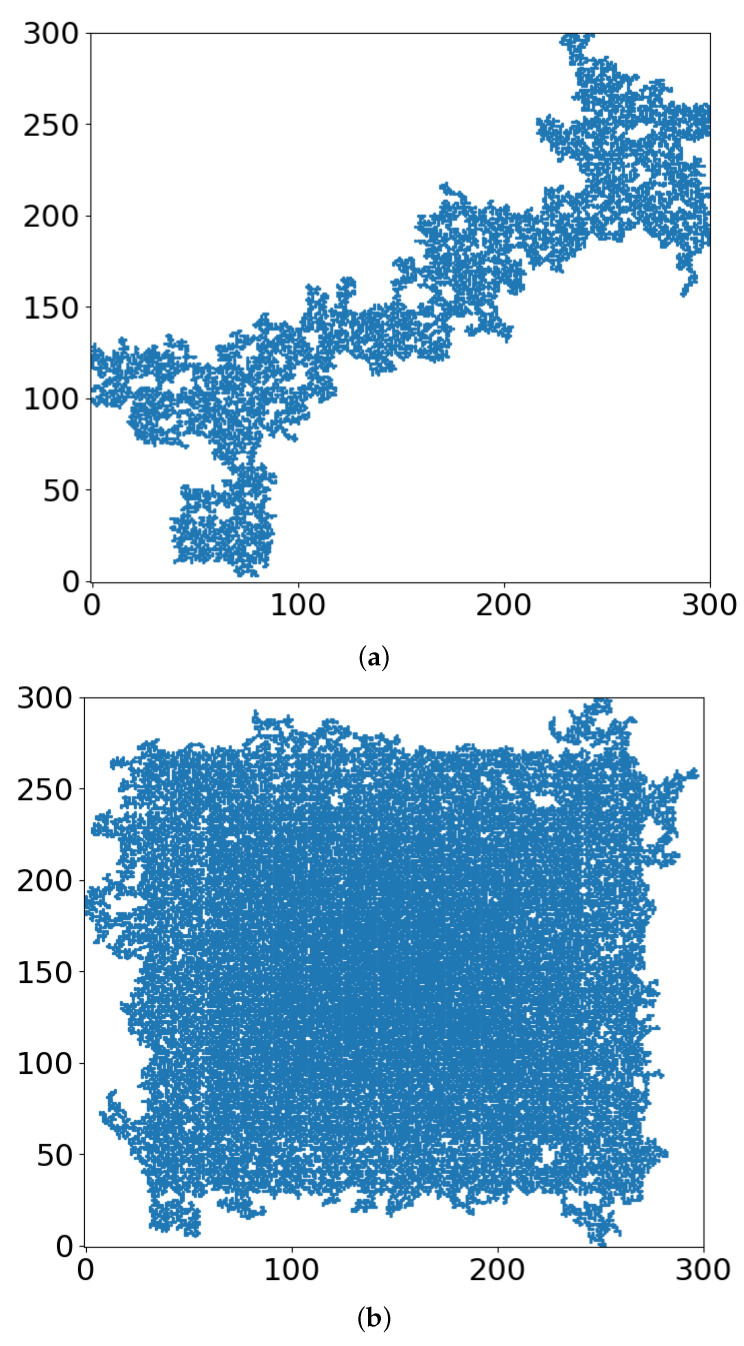
The largest cluster at the critical threshold for (**a**) r = 0, (**b**) r = 0.4.

**Figure 5:**
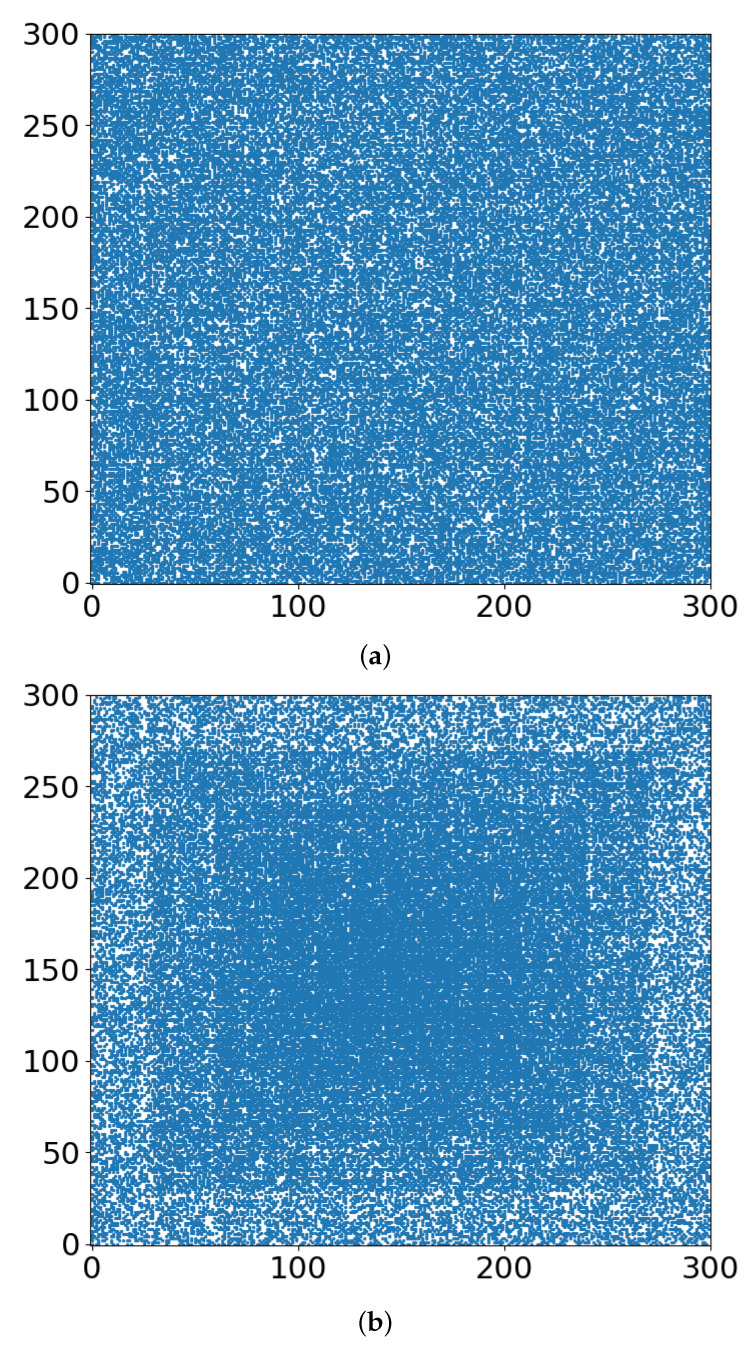
Occupied sites of the lattices at p = 0.5900 for (**a**) r = 0, (**b**) r = 0.4.

In Figure 3, we show the occupied sites of the lattice when the largest cluster percolates for r = 0 and r = 0.4. The largest cluster is presented in Figure 4, for r = 0 and r = 0.4. Finally, in Figure 5, we present the schematics of lattices just below the critical threshold, at p = 0.5900.

## 4. Discussion

In terms of radiation therapy and interference by the TME hypoxia, the following tentative conclusions can be drawn:

1. Whether the “occupied sites” (blue in Figure 3, Figure 4 and Figure 5) symbolize tumor cells or their TMEs, the percolation model simulations provide a useful intuitive picture of the consequences of different doses of radiation. At some dose, the tumor/TME network falls apart. This is a catastrophic, phase-transition-like, phenomenon, for which the percolation model was created historically. A small change (say an increase) of dose can have a large effect on therapy: the benefits of radiation are not linear with dose. Notably, its drawbacks are not discussed here.
2. With respect to the follow-up chemotherapy, to avoid the interference of TMEs’ acidity (acidosis) with drug sustainability, we are looking for guidance by the “unoccupied sites” (white in Figure 3 and Figure 4). To enable intact drug diffusion into the tumor, a clear (white) path would be helpful. An example is shown in Figure 4a, where it would be easy for the drug molecules to reach most of the remaining tumor network. However, note that the (white) “clear” areas are still full of smaller TME “clusters”, which are not shown. The analogy is a minefield where most mines were bombed out.
3. The “shell model” results, essentially those for r = 0, are highly illuminating. A look at Figure 3a and Figure 4a shows that the blue areas are now concentrated towards the center. The consequence would be that the chemotherapy drugs are prevented from reaching the tumor center, despite that the preceding radiotherapy is “poking holes” in the “minefield” of the acidic TMEs. This illustrates the major effect of the hypoxia heterogeneity, i.e., the limited oxygen diffusion into the tumor (and TME). We can see that even with partial oxygen diffusion (r > 0), only the tumor periphery may become amenable to successful follow-up chemotherapy, rather than the entire tumor, thus essentially enabling tumor regrowth.
4. Note that with just 41% of cell-kill (Figure 5a), the network of tumor cells (or TMEs) is all broken up into small (mathematically ”finite”) clusters. Medically, that is where chemotherapy should be effective, as the “minefield” has been rarefied and the drug molecules should be able to reach the tumor cells. Note also that just a “smidgeon” below 41%, with 40.7% cell-kill, we still find a connected network (Figure 4a, r = 0, where p_c_ is 0.5927). This illustrates the potentially sharp boundaries between sufficient and insufficient radiation dose.
5. On the other side, with hypoxia anisotropy, even with the same percentage of radiative cell-kill, the picture is totally different (Figure 5b). While at the periphery, the now-open (white in Figure 5b) channels are wider, the opposite happens towards the center. The channels do appear to be “closed” (blue in Figure 5b). The drugs will be blocked from reaching the tumor center’s cells, allowing for tumor regrowth. Therefore, a naïve expectation, say from Figure 2, where the critical concentration increases with r, which symbolizes hypoxia (i.e., an expectation that in the presence of hypoxia anisotropy less cell-kill will be required for tumor network break-up) is wrong. In actuality, the opposite seems to be the case. With 41% of cell-kill (Figure 5b), the tumor center appears to be dense enough to withstand any drug penetration of the TMEs. Thus, while the hypoxia anisotropy may assist chemotherapy at the periphery, it seems to resist it at the tumor center, with a bad resulting outcome of tumor regrowth.

Overall, the above points might help direct strategies for overcoming the above illustrations of radiotherapy interference by hypoxia and hypoxia heterogeneity: From having a patient breathe 100% O_2_, to targeted nanoparticles that provide the ingredients needed for producing ROS [13].

We realize that the studied square lattice model, while helpful mathematically, will hardly ever represent a real tumor. However, similar studies can be performed on realistic and even patient-specific tumor geometries. We envision such future studies that will enable personalized precision radiation oncology therapy.

## 5. Conclusions

Demonstrated above is the significant role that may be played by the tumor hypoxia distribution, i.e., hypoxia heterogeneity, on the effectivity of radiotherapy or combination radio- and chemotherapy. Thus, potential information on this hypoxia heterogeneity and its correlation with radiation treatment may be helpful, such as when obtained from in vivo photoacoustic imaging of xenografts [1].

## Figures and Tables

**Table 1:** Threshold values p_c_ as a function of r.

r	p_c_
0	0.592
0.1	0.608
0.2	0.642
0.3	0.676
0.4	0.709
0.5	0.738
0.6	0.764
